# Causes and Consequences of the Dysregulated Maternal Renin-Angiotensin System in Preeclampsia

**DOI:** 10.3389/fendo.2019.00563

**Published:** 2019-09-10

**Authors:** Eugenie R. Lumbers, Sarah J. Delforce, Anya L. Arthurs, Kirsty G. Pringle

**Affiliations:** ^1^School of Biomedical Sciences and Pharmacy, University of Newcastle, Newcastle upon Tyne, NSW, Australia; ^2^Priority Research Centre for Reproductive Sciences, University of Newcastle, Newcastle upon Tyne, NSW, Australia; ^3^Pregnancy and Reproduction Program, Hunter Medical Research Institute, Newcastle upon Tyne, NSW, Australia; ^4^Flinders Centre for Innovation in Cancer, Flinders University, Bedford Park, SA, Australia

**Keywords:** preeclampsia, utero-placental, renin-angiotensin system, miRNAs, angiotensin receptor autoantibodies, intrarenal angiotensin

## Abstract

A healthy pregnancy outcome depends on the activation of the renin-angiotensin-aldosterone system (RAAS) as a regulated, integrated response to the growing demands of the conceptus. Both the circulating RAAS and the intrarenal renin-angiotensin system (iRAS) play major roles in cardiovascular function and fluid and electrolyte homeostasis. The circulating RAAS becomes dysfunctional in preeclampsia and we propose that dysregulation of the iRAS plays a role in development of the clinical syndrome known as preeclampsia. Experimental studies in animals have shown that placental renin, when released into the maternal circulation, can cause hypertension. We postulate that abnormal placental development is associated with over-secretion of renin and other RAS proteins/angiotensin (Ang) peptides by the placenta/decidua into the maternal circulation. We hypothesise that this is because of increased shedding of exosomes and other placental particles into the maternal circulation that not only contain RAS proteins and peptides but also microRNAs (miRNAs) that target RAS mRNAs, and Ang II type 1 receptor autoantibodies (AT_1_R-AAs), that are agonists for, and have the same actions as, Ang II. As a result, there is both suppression of the circulating RAAS that is responsible for maintaining maternal homeostasis and activation of the iRAS. Together with altered vascular reactivity to Ang peptides, the iRAS causes hypertension, renal damage and secondary changes in the neurohumoral control of the maternal circulation and fluid and electrolyte balance, which contribute to the pathophysiology of preeclampsia.

## Introduction

Hypertensive diseases of pregnancy are the leading cause of maternal mortality in Latin America and the Caribbean and the second major cause of maternal mortality in developed countries. One of the most severe forms of hypertension in pregnancy is preeclampsia, which can lead to eclampsia and commonly results in maternal and fetal death (1, 2).

The placenta is quite rightly regarded as the cause of preeclampsia, as removal of the placenta is the only effective treatment. Thus, there has been a long-standing search for placental causes of preeclampsia.

A healthy pregnancy outcome depends on activation of the circulating renin-angiotensin-aldosterone system (RAAS) as a regulated, integrated response to the growing demands of the conceptus. The degree to which the maternal RAAS is activated in normal pregnancy is unique physiologically (3). Both the circulating RAAS and the intrarenal renin-angiotensin systems (iRAS) play major roles in regulating cardiovascular function and fluid and electrolyte homeostasis. If the peptides and proteins of the placental RAS (described below) escape into the maternal circulation, they disrupt both the circulating RAAS and the iRAS which become dysfunctional (4, 5).

We propose that abnormal or shallow placentation and/or reductions in uteroplacental exchange [such as occurs in experimental animal models that model human preeclampsia (6, 7)] cause dysregulated expression of the placental RAS. This leads leading to increased release into the maternal circulation from the placenta of RAS proteins and peptides (8) and microRNAs (miRNAs) that target the RAS. Experimental animal models have shown that human placental renin secretion can cause a preeclamptic-like syndrome. In preeclampsia, shallow placentation and alterations in uteroplacental perfusion are also accompanied by increased levels of angiotensin (Ang) II type 1 receptor (AT_1_R/*AGTR1*) autoantibodies (AT_1_R-AAs) that act as agonists on the AT_1_R (4, 9, 10). These molecules or the combination of these molecules could cause activation of the maternal iRAS (5) and failure of the circulating RAAS to respond to homeostatic demand.

**Figure 1:**
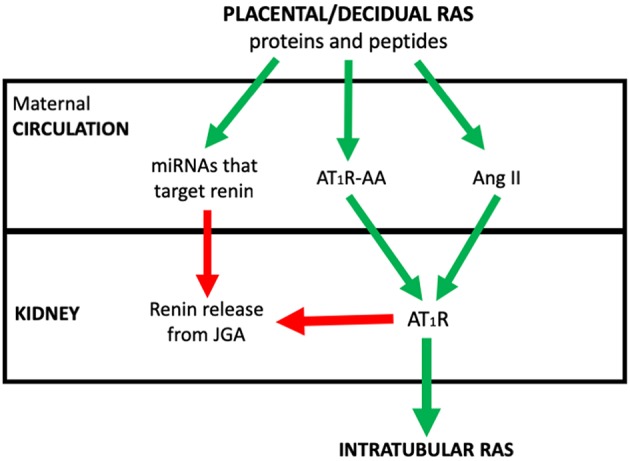
The postulated system via which placental/decidual renin-angiotensin system (RAS) proteins and peptides, miRNAs that target renin and auto-antibodies to the Angiotensin II type I receptor (AT_1_R-AA) interact with the juxtaglomerular (JGA) to control circulating active renin levels and the intratubular RAS. Red arrows denote where pathway is inhibitory. Green arrows are stimulatory.

The review concentrates on how abnormal placental development dysregulates the placental RAS and therefore the maternal circulating RAAS and iRAS, and describes the consequent changes in maternal physiology that underpin preeclampsia. The integration of these RASs in the aetiology of the syndrome of preeclampsia is outlined in Figure 1.

We describe the contributions of these various RASs to the clinical syndrome of preeclampsia and also help to explain why other conditions like chronic hypertension, renal disease, diabetes mellitus and some single nucleotide polymorphisms (SNPs) of angiotensinogen (AGT) are risk factors for preeclampsia (11).

The roles of other molecules generated by shallow placentation and/or reduced uteroplacental perfusion, such as anti-angiogenic factors, soluble fms-like tyrosine kinase-1 (sFlt-1) and soluble endoglin (sEng) and endoglin, in the pathogenesis of preeclampsia are not discussed.

In cases of abnormal placental development, we postulate, as described and referenced below that there is:

1. Over-secretion of renin and other RAS proteins/Ang peptides by the placenta/decidua (12, 13);
2. Excess shedding of exosomes and other placental particles into the maternal circulation (14, 15) that not only contain RAS proteins and peptides, but also miRNAs that target RAS mRNAs (16–18); and
3. Generation of AT_1_R-AAs that are agonists for, and have the same actions as, Ang II (4, 9; see **Figure 4**).

The consequences of unregulated release of RAS proteins and peptides, of miRNAs that target RAS mRNAs, and of AT_1_R-AAs into the maternal circulation are:

1. Suppression of the regulated and integrated maternal circulating RAAS (26), and activation of the iRAS (27, 28);
2. Reduction in uteroplacental blood flow to the feto-placental unit resulting in a feedback loop that causes further placental damage (7); and
3. Secondary changes in the neurohormonal control of cardiovascular and renal function (29, 30) that exacerbate the hypertensive effects of the overactive iRAS (see Figure 5).

This review describes the placental/decidual, circulating, and intrarenal RASs and changes in the activity of these systems in normal pregnancy. It contrasts these changes with the dysfunctional RASs seen in preeclampsia. It postulates that changes in preeclamptic RASs are caused by RAS proteins and peptides leaking from the placenta and secondary activation of the iRAS. Both systems dysregulate the circulating RAAS and could underlie the pathophysiology of preeclampsia. Because additional evidence is required to further test this hypothesis, the review also describes the research required to strengthen this hypothesis.

### The Unique Activation of the Circulating Maternal RAAS in Pregnancy (Figures 2, 3)

In pregnancy, extra salt must be retained to fill the greatly expanded cardiovascular system; this need is created not only by the growth and demands of the conceptus but also by the increased activity of other maternal organs responding to increasing fetal and placental metabolic demand. The cardiovascular system is expanded through the actions of relaxin (32), vasodilator peptides of the RAS, e.g., Ang-(1–7), downregulation of endothelial AT_1_R and upregulation of AT_2_R, vascular endothelial growth factor (VEGF), nitric oxide (NO), kallikrein-kinin, and prostanoids (3).

**Figure 2:**
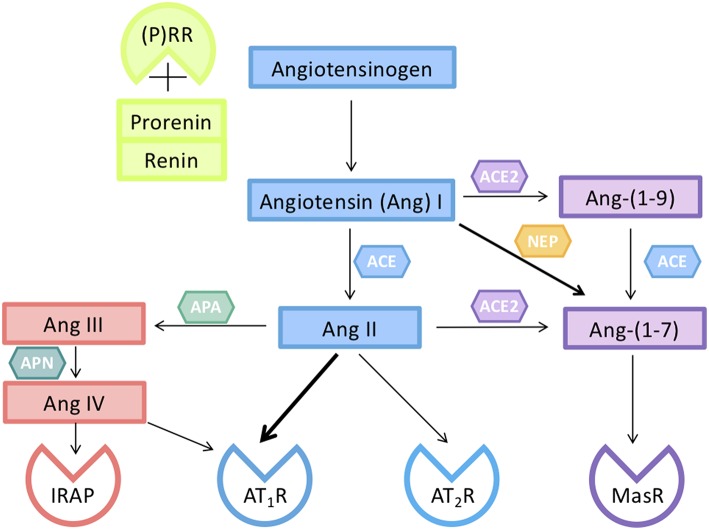
Components of the renin-angiotensin system (RAS) responsible for the activity of circulating and tissue RASs in pregnancy. Renin, or prorenin binding to the prorenin receptor ((P)RR), can cleave angiotensin I (Ang I) from Angiotensinogen (AGT). Ang I is converted to Ang II by a dipeptidyl carboxypeptidase, angiotensin converting enzyme (ACE). Ang II can act on either an Ang II type 1 receptor subtype (AT_1_R) or a type II receptor subtype (AT_2_R). The interaction of Ang II with its AT_2_R opposes the vasoconstrictor actions of Ang II mediated via its AT_1_R. Ang II can be converted by aminopeptidase A (APA) to Ang III which is converted to Ang IV. Ang IV acts on insulin regulated aminopeptidase (IRAP). Ang II is converted by the monopeptidyl carboxypeptidase ACE2 to Ang-(1–7), which acts on its own G-protein coupled receptor (MasR), and has actions similar to those of Ang II mediated via the AT_2_R. Ang peptides can often interact with different Ang receptors [see also (31)].

**Figure 3:**
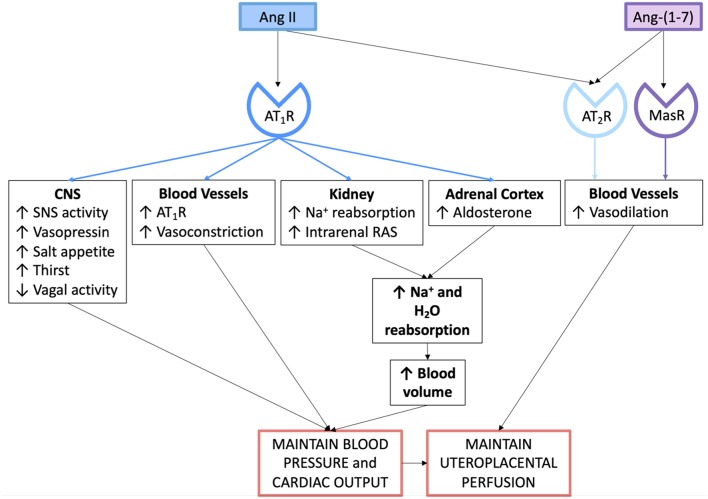
Actions of the angiotensin peptides on the cardiovascular system and on fluid and electrolyte homeostasis in normal pregnancy.

Thus, the capacitance of the cardiovascular system is increased. In early pregnancy, blood pressure falls and renal blood flow and glomerular filtration rate (GFR) increase by 50%. The maternal RASs (circulating and intrarenal) are activated so that salt and, therefore, water are retained. The increased demand for salt must occur despite this relaxin-induced increase in renal plasma flow and GFR, which promotes sodium excretion. Since the kidney plays the major role in regulating salt and water balance it has to increase the reabsorption of sodium. This depends on increased production of aldosterone and also on an activated iRAS.

A study carried out in pregnant Yanamamo women, who had no salt in their diet, demonstrates the importance of the RAAS in pregnancy (33). These women excreted ~1 mmol/day of sodium. Their urinary aldosterone levels were extraordinarily high (585 ± 46 ng/ml) compared with lactating Yanamamo women (46.2 ± 62.5 ng/ml) and non-pregnant (38.8 ± 31.4 ng/ml) women. Like urinary aldosterone, plasma renin activity in pregnant Yanamamo women was very much higher (25.6 ± 6.4 ng.ml^−^h^−1^) compared with lactating and non-pregnant Yanamamo women [5.0 ± 2.6 and 6.2 ± 4.1 ng.ml^−^h^−^, respectively, (34)]. These findings show that in response to pregnancy there is an increase in the activity of the RAAS over and above that required to maintain salt balance in lactating and non-pregnant women because after birth, both maternal blood volume and GFR are reduced.

The increased activity of the RAAS in normal pregnancy is accompanied by a reduction in the hypertensive actions of Ang II. This is because there is down-regulation of vascular AT_1_Rs resulting in a reduction in both vascular reactivity (35) and the vasopressor response to Ang II (36). The extent to which Ang-(1–7), NO and the presence of AT_2_Rs, which are highly expressed in pregnant uterine arteries (37), account for the blunted pressor response to Ang II and the extent to which Ang II's vasoconstrictor actions are offset by relaxin-induced changes in the maternal vasculature have not been quantitated.

### Components of the Placental, Circulating, and Intrarenal RASs (see Figure 2)

The major peptide of the RAS is Ang II, through which most of the known actions of the RAAS are mediated (Figure 3). More recently, Ang-(1–7), which has opposing actions to Ang II, has been found to play a significant role in human pregnancy and may well-contribute to the vasodilation characteristic of pregnancy, as well as having effects on renal vascular resistance and function (38).

The major pathway for the formation of Ang-(1–7) is through the conversion of Ang II via angiotensin converting enzyme 2 (ACE2, Figure 2). Ang I, Ang II, and Ang-(1–7) levels are all increased during normal pregnancy. It has been proposed that Ang-(1–7) could be used as a therapeutic for the treatment of preeclampsia as plasma levels are reduced in women with preeclampsia at term (39). However, the balance between Ang II and Ang-(1–7), rather than Ang-(1–7) deficiency alone may be involved in the aetiology of the syndrome. At 15 weeks' gestation, women who developed either preeclampsia or gestational hypertension and who were carrying a female fetus had higher Ang-(1–7) levels compared with BMI-matched controls (40). Preeclampsia occurs more commonly in women carrying a female fetus, despite the fact that at 15 weeks' gestation women carrying male fetuses who subsequently developed preeclampsia already had higher blood pressures (40). Since Ang-(1–7) is a downstream product of the cleavage of Ang II, the higher levels of Ang-(1–7) may be due to increased release of RAS proteins and Ang peptides into the maternal circulation from the placenta.

#### Renin

Renin is the active form of prorenin; the latter has no catalytic activity, unless it binds to the (pro)renin receptor ((P)RR) or is activated by low pH or by tissue proteases (41, 42). About 1% of prorenin can be spontaneously activated (43). Only the kidney secretes active renin in response to; reductions in renal perfusion pressure (the so-called renal baroreceptor), increased renal sympathetic nerve activity and/or changes in the concentration of sodium at the macula densa (MD), a specialised region of the very early distal convoluted tubule that forms part of the juxtaglomerular apparatus (JGA). Therefore, the secretion of active renin by the kidney is integrated into the neuroendocrine control of blood pressure and fluid and electrolyte balance. Tissue RASs constitutively only secrete prorenin. For these tissue RASs to generate Ang peptides, secreted prorenin must be activated as described above.

#### Angiotensinogen

Perhaps one of the most overlooked components of the circulating RAS is angiotensinogen (AGT). Circulating AGT is produced by the liver. Since the renin-AGT reaction is a first order reaction, it follows that levels of AGT influence the rate of production of Ang peptides. AGT production is stimulated by oestrogens and therefore its levels are strongly controlled by the ovary initially, and subsequently by oestrogen secretion from the feto-placental unit (44). SNPs in the AGT gene are associated with increased levels of AGT and an increased prevalence of preeclampsia (45).

Other factors that can affect the rate of production of Ang I from the renin-AGT reaction include the oxidation of AGT (which exposes the catalytic site) and soluble (P)RR (s(P)RR), which alters the affinity of renin for oxidised AGT. Levels of both oxidised and reduced AGT are increased in the plasma of women with preeclampsia as are levels of s(P)RR (46, 47).

#### Aldosterone

Aldosterone is secreted by the zona glomerulosa of the adrenal cortex. Ang II acting through the AT_1_R stimulates aldosterone release, which therefore promotes distal sodium reabsorption in response to homeostatic demand (48). Figure 3 summarises the mechanisms via which the circulating RAAS maintains blood pressure, blood volume and uteroplacental perfusion in normal pregnancy.

It is well-established that circulating levels of active renin, Ang II and aldosterone are suppressed in women with preeclampsia compared with levels measured in normotensive pregnant women (26, 49, 50).

## The Placental RAS

Placentation requires a complex interplay between proliferation of trophoblast cells and their invasion of the maternal decidua and spiral arterioles, which they plug so that in the first trimester the placenta normally develops in a low oxygen environment (51).

The placenta contains its own RAS (52). It is expressed from at least 6 weeks gestation and is most active early in gestation. Ang II, the major biologically active end-product of the RAS cascade, binds to placental AT_1_Rs and stimulates cell proliferation and migration, angiogenesis and trophoblast invasion (53–56). Later in pregnancy, the expression of the placental RAS is reduced, so that levels are lowest in near term placentae from uncomplicated pregnancies (52, 57).

### Regulation of the Placental RAS

**Figure 4:**
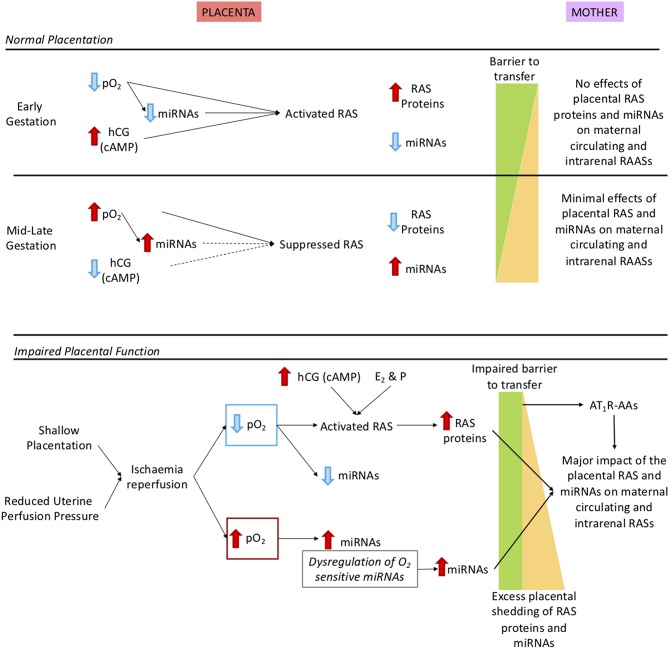
Description of how oxidative stress in the placenta caused by shallow placentation or reduction in uterine perfusion pressure could cause dysregulation not only of the placental RAS but also the maternal and intrarenal RASs. **Upper panel**: Normal control of the placental RAS in early and mid-late gestation. We have identified O_2_ regulated placental microRNAs (miRNAs) that target key RAS mRNAs. As well, expression of the placental RAS is regulated by hormones such as hCG, cAMP (19) and the combination of the steroid hormones oestrogen (E_2_) and progesterone [P; (20)]. In early gestation there is minimal shedding of placental particles and molecules (yellow triangle) and the total placental mass is very small but RAS targeting miRNAs that are oxygen-regulated are suppressed so even though the placental RAS is upregulated by low O_2_ no RAS proteins/peptides or O_2_ regulated miRNAs (17) are released. At 12 weeks, maternal blood flow increases and pO_2_ rises so that O_2_ regulated miRNAs are upregulated, leading to reduced placental RAS activity. Thus, despite the gestational increase in exosomal shedding (21), escape of placental RAS proteins and peptides into the maternal circulation is curtailed. **Lower panel**: The preeclamptic placenta. In preeclampsia there is placental ischaemia/reperfusion resulting from shallow placentation or a reduction in uterine perfusion pressure (6, 22, 23). Fluctuations in placental _p_O_2_ lead to dysregulation of O_2_ sensitive miRNAs and activation of the placental RAS. Together with excess placental shedding there is increased release into the maternal circulation of RAS proteins and peptides and of miRNAs that target the maternal RAS. These secretions are not regulated by homeostatic demand, creating a situation analogous to 2-Kidney 1-Clip hypertension or Ang II dependent hypertension; Ang II infusions have been used to model preeclampsia (8). There is also an added contribution of placental miRNAs known to target the RAS (24) that cause renin independent hypertension (25). Autoantibodies that acts as agonists to the AT_1_R (AT_1_R-AA) released from the decidua (4) have the same actions as Ang II. MiRNAs that target the maternal RAS and AT_1_R-AAs also suppress JGA-mediated renal renin release. Thus, there is a greater release of decidual/placental renin and Ang peptides into the maternal circulation, which lacks neurohumoral control so that the circulating system is not in accordance with homeostatic demand.

Some of the factors that regulate the placental RAS are shown in the upper panel of Figure 4. These include, but are not limited to, oxygen, human chorionic gonadotrophin (hCG), cyclic adenosine monophosphate (cAMP) and miRNAs. Exosomal shedding results in release of RAS proteins and peptides, as well as miRNAs, into the maternal circulation.

#### O_2_ Independent Regulation of the Placental RAS

It has long been known that hCG stimulates the secretion of prorenin from the placenta. Downing et al. first showed that β-hCG stimulates the secretion of prorenin from human placental explants (19, 58). They also showed that cAMP induced prorenin release from placental explants.

Wang et al. studied the effects of several putative regulators of prorenin expression and release in an immortalised first trimester extravillous trophoblast cell line (HTR-8/SVneo) (20). Cyclic AMP, global demethylation (using 5-aza-2-deoxycytidine), and the combination of oestrogen and medroxyprogesterone acetate all increased prorenin (*REN*) mRNA abundance and resulted in increased prorenin secretion. It is therefore likely that *in vivo* oestrogen, progesterone and hCG all play a role in regulating placental prorenin production. Perhaps the most interesting of these, with relation to preeclampsia, is hCG, as its levels are high in women who have preeclampsia (59) (particularly those with early-onset preeclampsia) and in the fetal circulation (60). Since hCG is known to stimulate both ovarian and placental prorenin production, its high secretion in early gestation could cause the excess release of renin into the mother at this time so priming dysregulation of the maternal RAAS and activation of the iRAS, which leads to the development of hypertension later in gestation.

#### O_2_ Dependent Regulation of the RAS; the Role of Placental miRNAs

Since the highest level of expression of the placental RAS occurs during the first trimester, when placental oxygen tensions are low (52), and its expression subsequently falls coincident with the onset of maternal blood flow (16, 52), we postulated that expression of the placental RAS could be regulated by oxygen sensitive miRNAs that target the RAS.

MicroRNAs repress gene expression by binding to target mRNAs. Each individual miRNA can potentially target ~60% of all genes (61–63). Likewise, a single gene can be regulated by multiple miRNAs. MicroRNAs have differing effects on cell development and growth depending on their physiological context and cognate mRNA (61, 64, 65).

MicroRNAs are short, single stranded non-coding RNAs approximately 22 nucleotides long. In the nucleus they are transcribed from DNA to primary miRNA (pri-miRNA), then cleaved by Drosha to form precursor miRNA (pre-miRNA) which is transported to the cytoplasm by Exportin-5. Dicer facilitates the formation of a miRNA duplex, which is subsequently unwound, with the 5′ strand becoming the mature miRNA. The protein Argonaute (Arg) 2 is added to the miRNA and the complex is loaded into RNA-Induced Silencing Complex (RISC).

Placental specific miRNAs, often from chromosome 19, are secreted into the maternal plasma during pregnancy (62, 66). Excess secretion of placental miRNAs as a result of increased shedding of placental microparticles that range in size from 10 nm to 100 μm (66) occurs when placentation is impaired [e.g., in preeclampsia (66, 67)]. Exosomes containing miRNAs are a component of placental debris (14). MicroRNAs are also found in the circulation, external to exosomes but bound to key proteins that form RISC such as Arg proteins (Arg 1 and 2), or to high-density lipoproteins [HDLs, (68)].

The inappropriate release of miRNAs into the maternal circulation in pregnancies complicated by placental insufficiency, resulting from oxidative stress could impact on maternal health by targeting maternal mRNAs (14, 67). Wang et al. using a microarray of 2006 placental miRNAs, showed that the expression of a number of placental miRNAs changed throughout gestation (16). Seventy-seven miRNAs had low levels of expression in placentae collected at 10 to 11 weeks' gestation compared with term placentae; 30 of these had the RAS as a putative target pathway. In placentae collected from 14 to 18 weeks' of gestation, expression of 48 miRNAs were suppressed relative to term placentae; 19 of these miRNAs had the RAS mRNAs as putative targets. The trans-gestational pattern of some of these miRNAs were confirmed using PCR (16).

Furthermore, the expression of some miRNAs that target the RAS were found to be enhanced in placentae collected from women with early and late onset preeclampsia compared with gestational age-matched uncomplicated placentae. In placentae from women with early-onset preeclampsia, miR-663 expression was increased; this miRNA also suppresses renal *REN* mRNA translation (16, 24). In late-onset preeclampsia, the placental expression of miR-378, which is predicted to target *REN* and *ACE* mRNAs, miR-514b-3p, predicted to target *AGT* and *AGTR1* mRNAs and miR-892c-3p, which targets *AGT* mRNA, were increased (16). Arthurs *et al*. showed that expression of these four miRNAs in HTR-8/SVneo cells was suppressed by incubation in 1% O_2_ and levels of their corresponding RAS target proteins (i.e., prorenin, ACE and AT_1_R) were enhanced (17). Lentiviral transfection of two miRNAs, miR-663 and miR-181a-5p into HTR-8/SVneo cells, knocked down expression of prorenin mRNA and protein (16). As well, HTR-8/SVneo cells transfected with mimics for miRNAs miR-181a-5p, miR-378, miR-181a-3p, and miR-663, all of which target *REN* mRNA, had significantly decreased expression of *REN* mRNA (18). These studies also showed that this downregulation of *REN* and *ACE* mRNA expression was associated with reduced trophoblast proliferation.

Therefore, the placental RAS is, in part, regulated by miRNAs and the expression of some of them is altered by oxygen and in placental pathologies. Furthermore, the ability of oxygen and miRNAs to regulate the abundance of placental RAS mRNAs and proteins alters placental development and function.

### Oxidative Stress, Hypoxia-Reoxygenation, and “Deportation of Placental Debris” in Preeclampsia

Throughout pregnancy the placenta sheds syncytiotrophoblasts, exosomes, and other microvesicles into the maternal circulation (21). These microvesicles contain a host of molecules that can interact with the maternal cardiovascular system and the kidneys, and alterations in their composition could be involved in the pathogenesis of abnormal pregnancy outcomes like preeclampsia, intrauterine growth restriction, and gestational diabetes (14, 15). The number of placental particles entering the maternal circulation increases with gestation (21).

Oxidative stress is associated with increased shedding of microvesicles (14) and is thought to be a major factor in the aetiology of preeclampsia (22), although these authors make a convincing case that ischaemia-reperfusion events may be more significant in that they induce apoptosis in the syncytiotrophoblast (23).

Inadequate remodeling of the maternal spiral arteries means that not only is there poor placental perfusion but also, the retained vascular reactivity of the vessels leads to episodes of intermittent contraction and relaxation. In late gestation, when placental pO_2_ falls from 60 to 40 mmHg, episodes of ischaemia-reperfusion will occur more frequently.

If one speculates that such a mechanism is responsible for excess shedding of placental material into the maternal circulation, then an excess of placentally produced peptides, proteins or miRNAs could enter the maternal circulation. Furthermore, fluctuating levels of O_2_ are likely to disrupt regulation of oxygen sensitive miRNAs (see above). As a result, excess shedding of placental material into the maternal circulation could release molecules that dysregulate maternal circulating and intrarenal RASs.

It is not known if exosomes and other microvesicles contain components of the placental RAS. It is however likely that soluble (P)RR (s(P)RR), generated from placental (P)RR, enters the maternal circulation in exosomes. Since (P)RR increases the catalytic activity of renin with oxidised AGT (46) and levels of both s(P)RR and oxidised AGT are increased in preeclampsia (46, 47), the increased release of s(P)RR could contribute to the dysregulation of maternal RASs in preeclampsia.

### Other Potential Disruptors of the Maternal RAS Released From the Placenta

Wallakat et al. first used a neonatal spontaneously contracting rat cardiomyocyte bioassay (9) to detect AT_1_R-AAs. Herse et al. found that plasma levels of these stimulatory antibodies (which are directed against the second extracellular loop of the AT_1_R) were increased in the plasma of women with preeclampsia [measured using the increase in the rate of beating of neonatal contracting cardiomyocytes (4)]. Normal plasma generated an increase in rate of 0.05 ± 0.4 bpm and preeclamptic plasma increased the rate by 17.5 ± 2.2 bpm (4). These autoantibodies are agonists for the AT_1_R, which was proven by showing that their actions were blocked by the AT_1_R blocking drug, losartan (4). Thus, AT_1_R-AAs could act in conjunction with any increases in conceptually released RAS proteins or peptides and stimulate Ang II-AT_1_R mediated pathways. Furthermore, because these AT_1_R-AAs, like Ang II, activate the AT_1_R receptor, they provide a simple explanation for the dissociation between the circulating and intrarenal RASs (see below). AT_1_R-AAs would suppress the release of renin into the maternal circulation while at the same time activating the iRAS by interacting with the AT_1_R in the proximal tubule (5).

### The Decidua as the Source of Extrarenal RAS Proteins/Peptides Compared With the Placenta

Shah et al. proposed that the high levels of plasma renin in women with preeclampsia is due to increased uptake by maternal vessels of increased amounts of decidual renin (12). Herse et al. did not however find increased expression of any components of the decidual RAS in preeclamptic decidua except the AT_1_R; even though they found high levels of AT_1_R-AAs (4). Despite the upregulation of AT_1_R in the decidua of preeclamptic women, levels of expression were still lower than those found in the placenta.

The decidua has the highest levels of expression of *REN* mRNA (52, 57) and secretes prorenin (69) so it is possible that it contributes prorenin and possibly active renin to the mother (as a result of activation by tissue proteases such as plasmin and cathepsin D).

There is sexual dimorphism in the expression of the decidual RAS. Maternal decidua collected from women carrying female fetuses express more prorenin mRNA and secrete more prorenin protein than decidua from women carrying male fetuses (69, 70) and the prevalence of early-onset preeclampsia is higher in pregnancies carrying female fetuses but not in late-onset preeclampsia. This sexual dimorphism is more marked in very preterm preeclampsia (71). In addition, in early gestation, as described above, maternal circulating Ang-(1–7) levels are higher in those women carrying female fetuses who subsequently develop preeclampsia or gestational hypertension (40) but women carrying male fetuses already have elevated arterial pressures. Thus, it is perhaps not surprising that there is some conflict concerning the impact of fetal sex on the prevalence of preeclampsia (72).

At term the placental RAS is normally suppressed (52), which explains in part why the decidual RAS is 4- to 5-fold more active than the placental RAS at this time. Decidual tissue consists of maternal decidualised stromal cells, which express RAS genes when endometrial stromal cells are stimulated to decidualise (73), fetal cells (mainly extravillous trophoblasts) and immune cells. Therefore, it could be a site of origin for RAS proteins and peptides entering the maternal circulation.

When female transgenic mice carrying the human *AGT* gene were mated with male mice carrying the human *REN* gene, they developed preeclampsia (hypertension and proteinuria) in mid-gestation (13). These animals had high circulating levels of human renin. This renin could only have been derived from the trophoblast and they provide conclusive evidence that placental trophoblasts (fetal cells) can secrete renin into the maternal circulation in amounts sufficient to cause a preeclampsia-like syndrome. This is similar to the uncontrolled release of renin by some renal renin-secreting tumours; in one case it was shown that the fall in blood pressure correlated with the post-operative decline in plasma renin after the tumour was removed (74).

Shah et al. postulated that increased renin release occurs from a poorly functioning placenta/decidua as a result of reductions in uteroplacental blood flow (12). They suggest this leads to excessive release of placental renin and Ang peptides into the mother creating a situation that is analogous to the effects of Ang II-dependent hypertension or two kidney one clip (2K-1C) hypertension (75). The overactivity of the iRAS in these models of hypertension is proposed to occur as a result of high levels of circulating Ang II (see below), which through its actions on the iRAS, causes sustained hypertension.

In established preeclampsia, circulating Ang II and aldosterone levels are suppressed (26). Therefore, the potential interactions of various placental molecules that act on the RAS need to be taken into consideration. It would be of value to determine the interactions between RAS proteins and peptides, AT_1_R-AAs and placental miRNAs that target the RAS in models like the Ang II-induced or 2K-1C animal models of hypertension.

## Renal Renin-Angiotensin Systems

### Juxtaglomerular Secretion of Active Renin

The kidney is the source of active renin in the maternal circulation. It is released from juxtaglomerular cells, which are granulated vascular smooth muscle cells lining the afferent arteriole. Its release is controlled by a renal baroreceptor, the sympathetic nervous system and the amount of sodium flowing past that region of the distal tubule, known as the MD, and which is located within the apex of a triangle formed by the afferent and efferent arterioles. This structure is known as the JGA. A low sodium concentration in the MD induces cyclooxygenase-2 (COX-2) resulting in increased prostaglandin production, which stimulates renin release (76). Through these regulatory mechanisms the activity of the circulating RAAS is controlled from the JGA, to maintain cardiovascular and fluid and electrolyte homeostasis. It is not surprising therefore, that active renin levels increase above mid-luteal levels as gestation proceeds and the demand for Ang II and aldosterone to maintain circulating blood volume outstrips the activation of the RAAS induced by oestrogen stimulated increases in AGT.

### Intrarenal RAS (iRAS)

As well as JGA-mediated control of renal renin, there is an iRAS that also plays a role in sodium homeostasis and hypertension through its effects on renal vascular resistance. This system is activated by Ang II interacting with the AT_1_R in the proximal tubule.

In Ang II-dependent hypertension and 2K-1C hypertension models, high levels of circulating Ang II stimulate the iRAS, creating an unstable feed forward mechanism resulting in intratubular production of Ang II (27, 28, 77, 78). AT_1_R-mediated Ang II uptake in the proximal tubule stimulates the synthesis/uptake of AGT in the proximal tubule resulting in the local production of Ang II and causing increased formation of Ang II in the distal segments of the nephron. Renin is produced in the distal nephron by the principal cells of the collecting ducts and catalyses the formation of Ang I from tubular AGT, generating Ang II. These increases in intrarenal, i.e., interstitial, and intratubular, Ang II concentrations occur even when plasma renin activity (PRA) is markedly suppressed (78). Accordingly, Ang II-infused rats develop high renal interstitial fluid concentrations of Ang II, which increase renal vascular resistance and reduce sodium excretion, so that there is resetting of the pressure-natriuresis relationship, described originally by Guyton (79). Coupled with the altered reactivity of peripheral resistance vessels, this causes hypertension (80). The augmentation of renal interstitial fluid Ang II is the result of an AT_1_R-mediated process that is dissociated from the circulating RAAS (5).

Furthermore, activation of the intrarenal AT_1_R is associated with downregulation of ACE2 and a reduction in intrarenal Ang-(1–7) levels. The kidney is a major site of production of Ang-(1–7), which has a number of effects on renal function. Ang-(1–7) acts as a vasodilator, lowers renal vascular resistance and protects against the development of Ang II-induced glomerulosclerosis (81).

## How Production of Placental RAS Proteins, Peptides, miRNAs That Target Maternal RAS mRNAs and AT_1_R-AAs Cause Dysregulation of the Maternal RAAS (Figure 1)

We suggest that increased production of placental prorenin and Ang peptides and recruitment of AT_1_R-AAs, stimulate the iRAS while continuing to suppress the maternally derived JGA-regulated circulating RAAS. Since AT_1_R-AAs stimulate AT_1_Rs they can further suppress renal renin secretion by the JGA, reducing renal renin release and Ang II production. Thus, the contribution of unregulated uteroplacental renin and Ang peptides to the maternal circulating RAS becomes greater. These RAS proteins and peptides are not controlled via the JGA. It should be noted that the pregnant uterus in the rabbit releases renin (82); as pregnancy continues it produces greater amounts of active renin relative to inactive renin (83) and it can be detected in the plasma of pregnant rabbits following bilateral nephrectomy (84).

Increased release of miRNAs that target the RAS, such as miR-181a, and others such as miR-663, which is upregulated in preeclamptic placentae, suppress renal renin (24). The presence of an excess of these and other miRNAs that target the RAS would also suppress the circulating RAAS. Plasma levels of miR-181a correlate directly with blood pressure and are associated with renin-independent hypertension (25).

As mentioned above, the interaction between renin and oxidised AGT, which is present in excess in the plasma of women with preeclampsia, is accelerated in the presence of s(P)RR, which is also increased in preeclampsia (47). It is known that (P)RR is expressed in the distal parts of the nephron where it plays a role in the acidification of urine and s(P)RR may also enhance the intratubular formation of Ang II (85).

Another “risk factor” in development of preeclampsia (11), namely the SNP M235T (rs699) in the AGT gene, which is associated with increased levels of AGT (86), could contribute to the hypertensinogenic profile of preeclampsia because intrarenal production of Ang II would be enhanced. Likewise, chronic renal disease, pre-existing hypertension and diabetes mellitus, through their interactions with the iRAS, increase the risk of developing hypertension in pregnancy (87).

Therefore, RAS proteins and peptides, and miRNAs and AT_1_R-AAs that target the RAS, should be measured in placental microvesicles from normal and abnormal placentae as they potentially have a role in dysregulating the maternal RASs, so contributing to the syndrome of preeclampsia.

### What Are the Consequences of Suppression of the Circulating RAAS and Activation of the Intrarenal RAS by RAS Proteins/Peptides, miRNAs, and AT_1_R-AAs (Figure 5)?

#### Hypertension

Activation of the iRAS and increased peripheral resistance (resulting from increased vascular reactivity), cause an increase in blood pressure despite downregulation of vascular AT_1_Rs and suppression of the circulating RAAS. This means that the neurohumoral control of the RAAS is impaired or lost.

**Figure 5:**
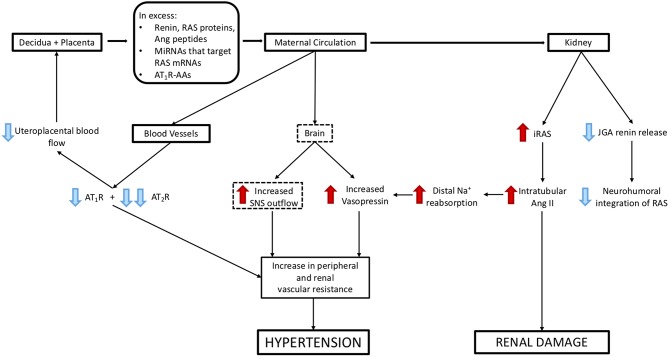
Mechanisms via which excess release of molecules from decidual/placental tissues that target the maternal kidney and cardiovascular system could cause hypertension and renal damage. Blue arrows represent suppression of maternal systems and receptors, red arrows represent stimulation of maternal systems and receptors. Dashed boxes highlight those pathways that have not been validated experimentally.

It has been claimed that intrarenal AT_1_Rs are increased in Ang II-induced hypertension. As stated above, renin and Ang II levels are depressed in the plasma of women with preeclampsia (26, 49). Ang-(1–7) levels are also depressed. It is not known whether this is as a result of decreased Ang II levels or is due to decreased production of Ang II in the kidney because ACE2 is suppressed when the iRAS is activated. However, the loss of Ang-(1–7) from the circulation along with the loss of vascular AT_2_Rs, which are downregulated in women with preeclampsia (4), means an overall reduction in those pathways in the RAS that offset the vasoconstrictor actions mediated by the AT_1_R.

#### Increased Vascular Reactivity Including the Uteroplacental Circulation

In pregnant sheep, high levels of Ang II downregulate vasodilator AT_2_Rs, which are located in abundance in pregnant uterine arteries. This leads to increased vascular reactivity and reductions in uteroplacental blood flow (88). When Ang II is infused into pregnant sheep, uteroplacental blood flow is maintained over the first 6 h of infusion; after 12 h however, it is reduced (89). This is associated with a loss of AT_2_Rs (88).

In pregnant sheep, clipping one renal artery also causes an immediate rise in blood pressure and after 24 h uteroplacental blood flow is reduced and the fetus is hypoxaemic (90). This can also be attributed to the downregulation of the AT_2_R so that only AT_1_R mediated vasoconstriction persists. Similar changes may occur in the human uteroplacental circulation. Downregulation of AT_2_Rs creates further placental damage so that a vicious cycle is set up which is associated with increased activity of the placental RAS (91). Uteroplacental hypoxaemia is responsible for the onset of preeclampsia like symptoms seen in animal models in which uteroplacental blood flow is reduced (6). Since AT_1_R-AAs have the same actions as Ang II it is likely that they also cause vasoconstriction within the uteroplacental circulation.

#### Sympathetic Activation

In normal pregnancy the gain of the cardiac baroreflex is attenuated but overall sympathetic activity is increased (92). There is some evidence suggesting that sympathetic activity is increased further in preeclampsia (93).

The reduction in the gain of the cardiac baroreflex in pregnancy has been attributed to a number of endocrine changes but Ang II does not appear to play a major role. However, in uncomplicated pregnancies, the central actions of increased Ang II levels could cause the observed increase in sympathetic outflow (92). Perhaps this interplay between Ang II and the central control of blood pressure is maintained or even enhanced in preeclampsia, because brain AT_1_Rs are not downregulated. It is unlikely however that AT_1_R-AAs can cross the blood brain barrier unless it is compromised.

#### Vasopressin

Vasopressin levels are increased in the plasma of women with preeclampsia and copeptin which is secreted *pari passu* with vasopressin has been suggested as a biomarker for preeclampsia (29, 94). Increased secretion of vasopressin is the appropriate response to activation of the iRAS because both proximal and distal tubular sodium reabsorption are stimulated. Distal tubular sodium reabsorption occurs in the water-impermeable distal nephron, as does aldosterone-induced sodium reabsorption. Therefore, to maintain osmotic balance, water reabsorption by the distal nephron must be increased. Vasopressin regulates the reabsorption of water by the distal nephron. Vasopressin is an extremely powerful vasoconstrictor that maintains blood pressure during dehydration (95) and has been used in animal models to cause coronary ischaemia. Therefore, its obligate secretion to maintain osmolar balance could contribute to the elevation of blood pressure and reductions in blood flow to vital organs seen in women with preeclampsia.

### Proteinuria

Ang II also causes glomerular damage (96), possibly by downregulating renal miRNAs that suppress calcium-calcineurin signalling and causing damage to renal podocytes (97). AT_1_R-AAs will likely have the same action. This leads to glomerular endotheliosis and escape of proteins into the urine. This may include AGT and in particular, oxidised AGT, as the source of proximal tubular AGT, however this is controversial. Surprisingly, unlike other causes of proteinuria, preeclampsia is associated with a reduction in the urinary excretion of AGT (98). Whether or not this is due to increased renal metabolism of AGT is only speculative.

## Conclusion

In conclusion, this review has examined the proposition that among the many molecules secreted by the placenta into the maternal circulation, and that could be involved in the pathogenesis of preeclampsia, are RAS proteins and peptides, miRNAs that target RAS mRNAs, and AT_1_R-AAs. Taken together, these molecules dysregulate the maternal RASs so that they are no longer regulated by the complex interplay of neural and endocrine pathways that ensure the kidney plays the correct role in regulating blood pressure and fluid and electrolyte balance in pregnancy. Dysregulation of maternal RASs has downstream effects that cause the clinical syndrome of preeclampsia.

## Author Contributions

EL wrote the manuscript. EL and KP are responsible for the intellectual concepts. SD, AA, and KP extensively reviewed the manuscript.

### Conflict of Interest Statement

The authors declare that the research was conducted in the absence of any commercial or financial relationships that could be construed as a potential conflict of interest.
